# Multimodal Image Fusion for X-ray Grating Interferometry

**DOI:** 10.3390/s23063115

**Published:** 2023-03-14

**Authors:** Haoran Liu, Mingzhe Liu, Xin Jiang, Jinglei Luo, Yuming Song, Xingyue Chu, Guibin Zan

**Affiliations:** 1 School of Data Science and Artificial Intelligence, Wenzhou University of Technology, Wenzhou 325000, China; 2 State Key Laboratory of Geohazard Prevention and Geoenvironment Protection, Chengdu University of Technology, Chengdu 610059, China; 3 The Engineering & Technical College of Chengdu University of Technology, Leshan 614000, China; 4 Sigray, Inc., Concord, CA 94520, USA

**Keywords:** Talbot-Lau interferometry, X-ray phase-contrast imaging, image fusion, non-subsampled contourlet transform, spiking cortical model

## Abstract

X-ray grating interferometry (XGI) can provide multiple image modalities. It does so by utilizing three different contrast mechanisms—attenuation, refraction (differential phase-shift), and scattering (dark-field)—in a single dataset. Combining all three imaging modalities could create new opportunities for the characterization of material structure features that conventional attenuation-based methods are unable probe. In this study, we proposed an image fusion scheme based on the non-subsampled contourlet transform and spiking cortical model (NSCT-SCM) to combine the tri-contrast images retrieved from XGI. It incorporated three main steps: (i) image denoising based on Wiener filtering, (ii) the NSCT-SCM tri-contrast fusion algorithm, and (iii) image enhancement using contrast-limited adaptive histogram equalization, adaptive sharpening, and gamma correction. The tri-contrast images of the frog toes were used to validate the proposed approach. Moreover, the proposed method was compared with three other image fusion methods by several figures of merit. The experimental evaluation results highlighted the efficiency and robustness of the proposed scheme, with less noise, higher contrast, more information, and better details.

## 1. Introduction

X-ray imaging techniques, such as mammography [1] and computed tomography (CT) [2], have become indispensable diagnostic tools for investigating the inner structure of materials. They can provide valuable information in many fields, from medical diagnosis to industrial inspection and security screening. Traditionally, the image contrast of these techniques depends on differences in X-ray attenuation. The attenuation contrast (μ) positively correlates with the material mass density (ρ) and atomic number (Z) ($\mu \propto \rho Z^{4}$), and negatively correlates with the X-ray energy (E) ($\mu \propto 1/E^{3}$) [3]. In principle, conventional X-ray attenuation-based imaging is ideal for materials with high absorption properties. However, the attenuation contrast becomes extremely poor without a significant increase in dose deposition, while low-Z materials are under investigation with high-energy X-rays.

Recently, X-ray grating interferometry (XGI) has been introduced to mitigate the inherent limitations of imaging low-Z materials using conventional X-ray imaging techniques. Because XGI is compatible with conventional low-coherence X-ray sources and detectors, it has become the most promising scheme for translating XGI into practice [4]. Moreover, XGI is a multi-contrast imaging technique, able to provide three physically different signals with complementary image contrast: attenuation contrast (AC), differential phase contrast (DPC), and small-angle scattering, also known as dark-field contrast (DFC) [5]. The phase signal can reveal differences between materials with similar absorption properties because it is highly sensitive to the electron density variations in the object. The scattering signal can access unresolved structural variations of the sample in the micrometer scale, which is beyond the system resolution. Many studies have demonstrated that both differential phase and scattering modalities were able to offer valuable information in addition to conventional attenuation contrast, including clinical applications such as mammography [6,7] and lung imaging [8,9] in addition to non-destructive testing [10] and material science in industrial settings [11]. The scattering signal, in particular, has piqued the attention of researchers because of its effectiveness in offering quantitative or inaccessible structural information in radiographic applications [12,13,14].

Adding two more informationally-complementary contrasts to the conventional attenuation contrast can enrich the information access channels. However, the three output images represent morphological features of an object with different physical properties, which can significantly enhance the complexity of interpretation and burden a physician. Image fusion could combine the tri-contrast modalities into a single integrated image, making analysis and diagnosis less cumbersome. The simultaneous acquisition of the tri-contrast images circumvents the preregistration process for image fusion because the retrieved AC, DPC, and DFC images are temporally and spatially registered. This could be particularly advantageous for reducing artifacts in the fusion procedure and conserving the reliability of the acquired information.

Tri-contrast image fusion methods have been developing over the past few decades. Ewald Roessl et al. presented, in 2012, an image fusion algorithm to combine AC and DPC based on an assumption of a simple scaling law [15]. However, the DFC signal was not considered for the procedure. Z. Wang et al. proposed a tri-contrast fusion method based on multiple resolutions in 2013 [16]. It successfully transformed details from the original images to the fusion results. However, the study lacked objective measurements to evaluate the method’s performance. Felix Scholkmann et al. proposed an image denoising, fusion, and enhancement scheme in 2014 [17]. It had pleasing results in both dental and breast imaging applications because it introduced pre-denoising and after-enhancement. However, the fusion rule of their scheme was unable to process three input images simultaneously, making it unsuitable for trimodal application. Eduardo Coello et al. introduced a Fourier domain framework for XGI fusion in 2017 [18]. The fusion results contained abundant diagnostic features and details, attributed to the full utilization of complementary information from three XGI channels by the Fourier transform. However, they did not compare it with other image fusion algorithms.

In this work, an XGI fusion scheme, based on the non-subsampled contourlet transform (NSCT) and the spiking cortical model (SCM), was proposed to solve several drawbacks of the current tri-contrast image fusion methods mentioned above. This scheme was able to process tri-contrast images from three channels of XGI simultaneously. It incorporated the pre-denoising processes of XGI outputs, the fusion process (based on NSCT-SCM), and the post-enhancement process of the fusion results. The proposed fusion algorithm was able to extract fine details and essential information from the tri-contrast images of XGI, presenting them in a final fused image with high contrast and low noise. The similarity between the fusion result and AC, DPC, and DPC channels of XGI was modulated by several tannable parameters, facilitating the easy realization of prior knowledge and preferences for particular channels. 

Moreover, the proposed fusion scheme was compared with the three XGI fusion methods mentioned above, i.e., the work of Felix Scholkmann et al. [17], the conventional NSCT image fusion algorithm, and the conventional NSCT-pulse-coupled neural network (PCNN) image fusion algorithm. The comparison was carried out in both subjective and objective evaluations. Objective measures incorporated edge strength (ES), spatial frequency (SF), standard deviation (SD), entropy (H), feature mutual information (FMI), feature similarity index measure (FSIM), fusion factor (FF), structural similarity index measure (SSIM), and power spectral density (PSD). Experimental results demonstrated the robustness and effectiveness of the proposed multimodal image fusion scheme.

The rest of this study was organized as follows: the basic principles of XGI fusion, NSCT, and SCM were presented in Section 2; the proposed NSCT-SCM XGI fusion scheme was illustrated in Section 3; the introduction of objective evaluation criteria was presented in Section 4; the experimental analysis of the proposed method was presented, together with the comparison with the other three algorithms for XGI fusion, in Section 5; and conclusions were drawn in Section 6.

Contributions of this study:

(1) drawbacks of image fusion methods in the XGI were analyzed;
(2) an image fusion scheme based on NSCT-SCM for the XGI was proposed;
(3) a tunable sub-band coefficient selection strategy was proposed to serve special requirements for the XGI fusion;
(4) the proposed NSCT-SCM image fusion scheme was applied to XGI data of frog toes and compared with current fusion methods in the XGI fusion field, exhibiting state-of-the-art performance.

## 2. Materials and Methods

### 2.1. Image Fusion for X-ray Grating Interferometry

X-ray grating interferometry simultaneously retrieves three complementary signals: AC, DPC, and DFC channels. Among these signals, AC represents the attenuation of the X-ray intensity; therefore, it provides the same information as conventional X-ray imaging, presenting it in the form of an X-ray absorption coefficient. DPC, on the other hand, is presented in the form of a refraction index, which relates to the X-ray’s local deflection. Finally, DFC is defined by the small-angle X-ray scattering at sub-pixel structures, presenting detailed information that would not be easily visible in the previous channels.

In XGI image fusion, high-frequency components of images from DPC and DFC are selected to provide greater features and details. At the same time, low-frequency components of the image from AC are preferred because of an intrinsic principle of conventional X-ray methods: making images easy for doctors or radiologists to read [18]. In addition, because the three pictures from XGI are retrieved simultaneously from the same direction by the same sensor, there is no need for additional image registration.

### 2.2. Non-Subsampled Contourlet Transform

**Figure 1:**
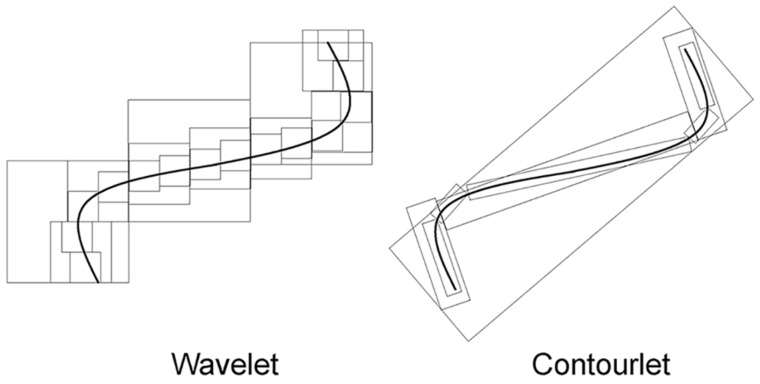
Describing a smooth contour by two different schemes.

Minh N. Do and Martin Vetterli proposed the contourlet transform (CT) in 2005 [19]. The following analogy demonstrates the advantages of CT; imagine there are two painters, one using a wavelet style and the other using a contourlet style. Both plan to paint a natural scene. Each painter increases the resolution of their painting from coarse to fine, step by step. When painting a smooth contour, as shown in Figure 1, the wavelet-style painter can only use square-shaped brush strokes along the contour [20]. He uses different-sized brush strokes, corresponding to the multiresolution structure of wavelets [21,22]. As the resolution grows finer, it becomes apparent that this painter needs to use a significant number of fine dots to describe the contour. However, the contourlet-style painter, in the same scenario, effectively and efficiently maintains the smoothness of the contour, attributed to their using brushstrokes with different elongated shapes, following the directions of the contour. This analogy gives a clear view of the advantages of the CT compared with the wavelet: the CT decomposes an image following its contour, which makes it less computationally complex than the wavelet.

**Figure 2:**
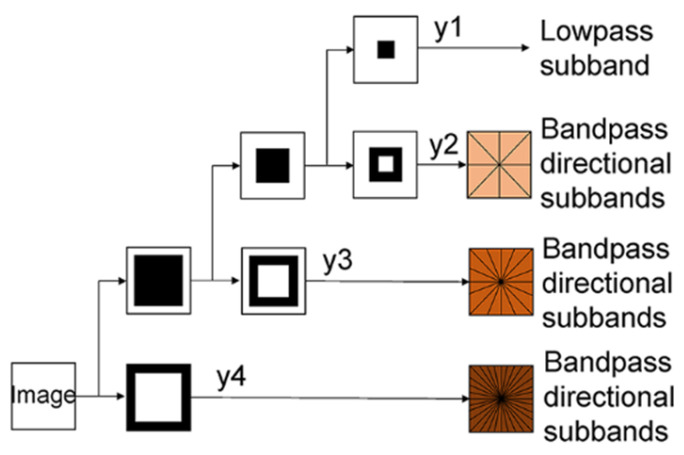
Image decomposition process of NSCT.

Derived from CT, NSCT is a multi-directional, multi-scale transform that can analyze detailed information in an image [23,24]. It uses the non-subsampled pyramid filter bank (NSPFB) and the non-subsampled directional filter bank (NSDFB), and thus, it achieves the shift-invariance property. First, the input image is decomposed into two parts by NSPFB: high-pass and low-pass sub-bands. Then, the high-pass sub-band is further decomposed into serval directional sub-bands by the NSDFB. Meanwhile, the low-pass sub-band continues to implement the above decomposition as a new input. As shown in Figure 2, when the decomposing process is done, one low-pass sub-band and serval high-pass directional sub-bands are obtained from an original input image. Note that the size of each sub-band is the same as that of the original image because there is no sampling operation. Moreover, NSCT has a redundancy, given by $R=\sum_{j=0}^{j}2^{l_{j}}$, where $2^{l_{j}}$ is the number of directions at scale j.

### 2.3. Spiking Cortical Model

The spiking cortical model [25] is a modified model, based on Eckhorn’s neural network, that uses physiology as inspiration [26]. It has fewer parameters and better accuracy than the original model. Its time matrix can be recognized as a subjective, human sense of stimulus intensity. As a result of these physiology-inspired neural networks’ outstanding ability to extract dynamic information inside multi-dimensional signals, they have been widely used in numerous fields. Instances include feature extraction [27], pulse shape discrimination [28,29,30], image encryption [31], and image segmentation and fusion [32,33].

Considering a biological neuron in a resting state, the membrane potential of this neuron is directly charged by external stimulus. Meanwhile, this membrane potential is modulated by the postsynaptic action potential of its neighboring neurons. In comparison, the membrane potential of SCM is similar to the aforementioned biological neural activity. The membrane potential of neurons in the SCM is calculated by combining the external stimulus and the neighboring modulation. A neuron in the SCM is fired and produces a spike when its neural membrane potential rises over its threshold. The threshold is dynamic, constantly changing under the influence of membrane potential states. Based on the characteristics mentioned above, the mathematical formulae of the SCM [25] can be written as follows:

(1) $$U_{ij}\left(n\right)=fU_{ij}\left(n-1\right)+S_{ij}\left(1+\beta \sum_{kl}W_{ijkl}Y_{kl}\left(n-1\right)\right),\;$$

(2) $$Y_{ij}\left(n\right)=\left\{\begin{array}{c} \;1,\;if\;\frac{1}{1+exp\left(-\left(U_{ij}\left(t+\Delta t\right)-\theta_{ij}\left(t+\Delta t\right)\right)\right)}>0.5\\ 0,\;otherwise \end{array}\right.,$$

(3) $$\Theta_{ij}\left(n\right)=g\Theta_{ij}\left(n-1\right)+hY_{ij}\left(n\right),$$

where each neuron is denoted by a coordinate $\left(i,j\right)$; coordinate $\left(k,l\right)$ represents one of the neighboring neurons of the central neuron located at $\left(i,j\right)$; $U_{ij}\left(n\right)$ is the membrane potential of a neuron located at $\left(i,j\right)$ when the iterative count is n; $S_{ij}$ is the external stimulus; $\Theta_{ij}$ is the dynamic threshold; $Y_{ij}\left(n\right)$ is the output action potential (spike); the convolution of W and Y stands for the modulation on the center neuron, located at the $\left(i,j\right)$ coordinate by its neighborhood neurons; W is the synaptic weighted matrix; β is the linking strength coefficient; f denotes the attenuation constant of the membrane potential which defines the gathering speed of it; and g represents the threshold’s attenuation constant, controlling the relative refractory period (i.e., the difficulty of activating peripheral neurons). Finally, h indicates the absolute refractory period, which prevents a neuron that has just been fired from immediately being reactivated again.

## 3. NSCT-SCM Fusion Scheme

**Figure 3:**
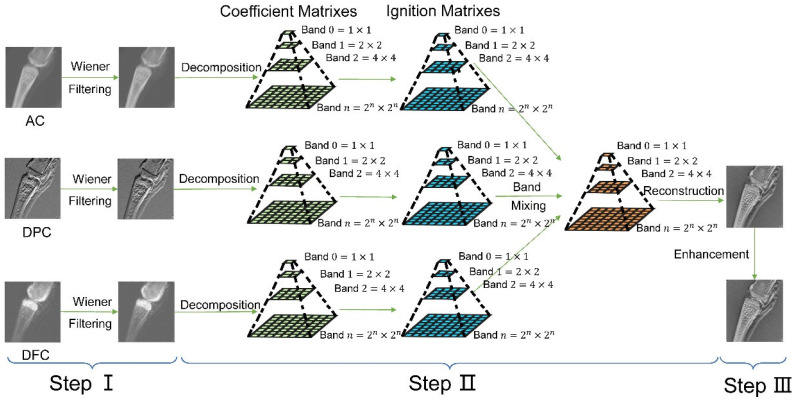
Principle of the NSCT-SCM XGI fusion scheme. Step I: Images are denoised using Wiener filtering. Step II: Images are decomposed into coefficient matrixes using NSCT. Then, the coefficient matrixes are proposed by SCM, outputting ignition matrixes. Finally, band mixing is implemented (three coefficient matrixes are fused into one coefficient matrix based on a coefficient selection algorithm designed on the basis of ignition matrixes), and the fused image is obtained by reconstructing the fused coefficient matrix. Step III: The fused image is enhanced to generate the final output image.

The proposed image fusion scheme incorporated three steps: (i) denoising all three input images (AC, DPC, and DFC) using adaptive Wiener filtering, (ii) implementing the NSCT-SCM based image fusion algorithm to the input images, and (iii) enhancing the output fused image using contrast-limited adaptive histogram equalization (CLAHE), adaptive sharpening (AS) and gamma correction (GC). The principle of the NSCT-SCM XGI fusion scheme is introduced in Figure 3.

### 3.1. Step 1. Image Denoising Based on Wiener Filtering

To obtain better quality raw images, the adaptive Wiener filter was applied to reduce the noise from an image while preserving the high-frequency information and edge features. The sizes of each input image are denoted by M×N; the AC, DPC, and DFC images are represented by $I_{AC}=\left\{I_{AC}\left(i,j\right)\right\}$, $I_{DPC}=\left\{I_{DPC}\left(i,j\right)\right\}$, and $I_{DFC}=\left\{I_{DFC}\left(i,j\right)\right\}$, respectively, where i=1,2,⋯,M and j=1,2,⋯,N. The image $I^{D}$ obtained after Wiener filter processing is expressed as follows [34]:

(4) $$I^{D}\left(i,j\right)=m+\frac{\sigma^{2}-v^{2}}{\sigma^{2}}\left(I\left(i,j\right)-m\right),\;$$

(5) $$m=\frac{1}{XY}\sum_{i=1}^{X}\sum_{j=1}^{Y}I\left(i,j\right),$$

(6) $$\sigma^{2}=\frac{1}{XY}\sum_{i=1}^{X}\sum_{j=1}^{Y}I^{2}\left(i,j\right)-\mu^{2},$$

where, m stands for the local mean, $\sigma^{2}$ denotes the local variance, and $v^{2}$ denotes the noise variance; X and Y are manual parameters which define the processing window size in the to-be-processed image I; and $\mu^{2}$ represents the average noise variance. After implementing adaptive Wiener filtering to images AC, DPC, and DFC, the output images are presented as $I_{AC}^{D}$, $I_{DPC}^{D}$ and $I_{DFC}^{D}$.

### 3.2. Step 2. NSCT-SCM XGI Fusion Algorithm

In this step, three images ($I_{AC}^{D}$, $I_{DPC}^{D}$ and $I_{DFC}^{D}$) were fused into one image, $I_{F}^{D}$.

1. First, the NSCT was implemented to the $I_{AC}^{D}$, $I_{DPC}^{D}$ and $I_{DFC}^{D}$ obtaining images’ high-frequency coefficients ($H_{AC}^{D,n}$,$\;H_{DPC}^{D,n}$ and $H_{DFC}^{D,n}$) and low-frequency coefficients ($L_{AC}^{D}$,$\;L_{DPC}^{D}$ and $L_{DFC}^{D}$), where n denotes the index of high-frequency coefficients, because multiple high-frequency coefficients are decomposed from a single image. Note that the size of each coefficient obtained from NSCT was the same as the input images, M×N in this case. Additionally, although only one low-frequency coefficient could be obtained from the NSCT process, multiple high-frequency coefficients could be gained from the NSCT of a single image, depending on the decomposition levels of NSDFB and NSPFB.
2. Second, high-frequency coefficients and low-frequency coefficients were fed into the SCM, generating the state of the firing of each coefficient ($T_{AC}^{D,n}$, $T_{DPC}^{D,n}$, or $T_{DFC}^{D,n}$ for the high-frequency coefficient and $T_{AC}^{D,L}$, $T_{DPC}^{D,L}$, or $T_{DFC}^{D,L}$ for the low-frequency coefficient), i.e., the ignition matrix. Each ignition matrix has the same size as its input coefficient, which was M×N in this case.
3. Two separate fusion rules were provided for high-frequency and low-frequency coefficients because of the need to preserve details and features in the high-frequency sub-band and keep the low-frequency part of the fused final image closer to the AC image. It is easier for doctors or radiologists to analyze a fused tri-contrast image when its low-frequency sub-band is close to that of the AC channel. Under this condition, the final fusion results will generally resemble the effects of traditional absorption-based tomography while containing complementary information of DPC and DFC channels.
  For the low-frequency coefficients:
  (7) $$L_{F}^{D}\left(i,j\right)=\left\{\begin{array}{c} L_{AC}^{D}\left(i,j\right),\;a\cdot T_{AC}^{D,L}\left(i,j\right)>\left(1-a\right)\cdot T_{DPC}^{D,L}\left(i,j\right)\;and\;\left(1-a\right)\cdot T_{DFC}^{D,L}\left(i,j\right)\\ L_{DPC}^{D}\left(i,j\right),\;\left(1-a\right)\cdot T_{DPC}^{D,L}\left(i,j\right)>a\cdot T_{AC}^{D,L}\left(i,j\right)\;and\;\left(1-a\right)\cdot T_{DFC}^{D,L}\left(i,j\right)\;\\ L_{DFC}^{D}\left(i,j\right),\;\left(1-a\right)\cdot T_{DFC}^{D,L}\left(i,j\right)>a\cdot T_{AC}^{D,L}\left(i,j\right)\;and\;\left(1-a\right)\cdot T_{DPC}^{D,L}\left(i,j\right)\;, \end{array}\right.$$
  where $L_{F}^{D}$ is the fused low-frequency coefficient and a is a tunable parameter that determines the similarity between the fused image and the AC image; the larger the value of a, the closer the fused image will be to the AC image.
  For the high-frequency coefficients:
  There were a total of 7 possible values for $H_{F}^{D,n}\left(i,j\right)$: (1) $H_{F}^{D,n}\left(i,j\right)=b\cdot H_{AC}^{D,n}\left(i,j\right)+c\cdot H_{DPC}^{D,n}\left(i,j\right)+d\cdot H_{DFC}^{D,n}\left(i,j\right)$; (2) $H_{F}^{D,n}\left(i,j\right)=H_{AC}^{D,n}\left(i,j\right)$, (3) $H_{F}^{D,n}\left(i,j\right)=H_{DPC}^{D,n}\left(i,j\right)$; (4) $H_{F}^{D,n}\left(i,j\right)=H_{DFC}^{D,n}\left(i,j\right)$; (5) $H_{F}^{D,n}\left(i,j\right)=\left(H_{AC}^{D,n}\left(i,j\right)+H_{DPC}^{D,n}\left(i,j\right)\right)/2$; (6) $H_{F}^{D,n}\left(i,j\right)=\left(H_{AC}^{D,n}\left(i,j\right)+H_{DFC}^{D,n}\left(i,j\right)\right)/2$; and (7) $H_{F}^{D,n}\left(i,j\right)=\left(H_{DPC}^{D,n}\left(i,j\right)+H_{DFC}^{D,n}\left(i,j\right)\right)/2$. The programming idea of the high-frequency fusion rule was such that we set a threshold T for the comparison of ignition results $T_{AC}^{D,L}$, $T_{DPC}^{D,L}$, and $T_{DFC}^{D,L}$. This comparison measured whether the information of a pixel coming from a single channel was significant enough to replace the others or whether a weighted average of the information of two or three channels was required. To be specific, when one channel was significantly larger than others, we chose the coefficient from this channel as the value of the $H_{F}^{D,n}\left(i,j\right)$ directly. When two were significantly larger than the rest, we took the average as the value of the $H_{F}^{D,n}\left(i,j\right)$. When no channel was significantly larger than the others, we weighted averaged the value of all three channels as the value of the $H_{F}^{D,n}\left(i,j\right)$ by the weight factors b, c, and d. A detailed fusion scheme of high-frequency coefficients is presented in the Supplemental Information, Section S1.
4. Finally, the inverse NSCT was implemented with respect to the low-frequency coefficients $L_{F}^{D},$ as well as the high-frequency coefficients $H_{F}^{D,n}$, obtaining the fused image $I_{F}^{D}$.

### 3.3. Step 3. Image Enhancement Using CLAHE, AS, and GC

Contrast-limited adaptive histogram equalization (CLAHE), adaptive sharpening (AS), and gamma correction (GC) were introduced to improve the image quality by Felix Scholkmann et al. [17]. This scheme was convenient to implement and was able to facilitate the output of better-quality images. Although it could enhance the image contrast and sharpness, it could not add further information to the fused image from the original AC, DPC, and DFC channels. Its application incorporated the following steps:

1. The image $I_{F}^{D}$ was first processed by CLAHE [35], which divided it into small tiles and changed the histogram of these tiles to enhance their contrast. Additionally, a clipping limit needed to be applied to the aforementioned processing, aiming to prevent excessive noise in the image. Bilinear interpolation was implemented on the tiles to avoid image discontinuities. After the implementation, the processed image $I_{F}^{En_{1}}$ was obtained.
2. Second, $I_{F}^{En_{1}}$ was sharpened by the AS method, mathematically given by:
  (8) $$I_{F}^{En_{2}}\left(i,j\right)=I_{F}^{En_{1}}\left(i,j\right)-C\nabla^{2}\;I_{F}^{En_{1}}\left(i,j\right),\;$$
  (9) $$\nabla^{2}\;I_{F}^{En_{1}}\left(i,j\right)=\frac{\partial^{2}I_{F}^{En_{1}}\left(i,j\right)}{\partial i^{2}}+\frac{\partial^{2}I_{F}^{En_{1}}\left(i,j\right)}{\partial j^{2}},$$
  where
  (10) $$\frac{\partial^{2}I_{F}^{En_{1}}\left(i,j\right)}{\partial i^{2}}=I_{F}^{En_{1}}\left(i+1,j\right)+I_{F}^{En_{1}}\left(i-1,j\right)-2I_{F}^{En_{1}}\left(i,j\right),$$
  (11) $$\frac{\partial^{2}I_{F}^{En_{1}}\left(i,j\right)}{\partial j^{2}}=I_{F}^{En_{1}}\left(i,j+1\right)+I_{F}^{En_{1}}\left(i,j-1\right)-2I_{F}^{En_{1}}\left(i,j\right),$$
  where C is the weighting factor adaptively determined by calculating the image entropies with many values of C and finding the $C_{max}$ value, i.e., when the maximum entropy was obtained. The final C was calculated by $C=C_{max}\left(argmax\left(H\right)\right)/\alpha$, where α is a constant to preserve the image becoming over-sharpened, with a fixed value of 3, empirically given by Felix Scholkmann et al. in their work [12]. After the aforementioned process, the image $I_{F}^{En_{2}}$ was obtained.
3. Finally, in the GC step, the image $I_{F}^{En_{2}}$ was enhanced by a sigmoid function, denoted as:
  (12) $$I_{F}^{En_{3}}=\frac{1}{1-exp\left[\lambda_{1}\left(\lambda_{2}-I_{F}^{En_{2}}\right)\right]},$$
  where $\lambda_{1}$ and $\lambda_{2}$ are two manually tunable parameters.

## 4. Measures of the Fusion Performance

With regard to fusion performance evaluation, there are two kinds of evaluation strategies: subjective and objective evaluations. Subjective evaluation is difficult to reproduce and highly dependent on the evaluators’ experience, making the evaluation results unstable and difficult to quantify. In this study, we chose the objective evaluation method as the primary method by which to compare the results of the proposed fusion scheme with the other fusion algorithms. Several performance measures were implemented for the fusion results in our experiment, as follows:

1. Edge strength (ES) [36] stands for the relative amount of edge information transferred from the input images ($I_{AC}$, $I_{DPC}$, and $I_{DFC}$) into the fused result $I_{F}$, denoted as:
  (13) $$ES=\frac{\sum_{i=1}^{M}\sum_{j=1}^{N}\left[ES_{AC,\;F}\left(i,j\right)w_{AC}\left(i,j\right)+ES_{DPC,\;F}\left(i,j\right)w_{DPC}\left(i,j\right)+ES_{DFC,\;F}\left(i,j\right)w_{DFC}\left(i,j\right)\right]}{\sum_{i=1}^{M}\sum_{j=1}^{N}\left[w_{AC}\left(i,j\right)+w_{DPC}\left(i,j\right)+w_{DFC}\left(i,j\right)\right]},\;$$
  where $w_{AC}\left(i,j\right)$, $w_{DPC}\left(i,j\right)$, and $w_{DFC}\left(i,j\right)$ are the weights, assigned to edge preservation values $ES_{AC,F}\left(i,j\right)$, $ES_{DPC,F}\left(i,j\right)$, and $ES_{DFC,F}\left(i,j\right)$ for $I_{AC}$, $I_{DPC}$, and, $I_{DFC}$, respectively. This edge preservation value was calculated through a Sobel edge operator, detailed information of which can be found in [36]. The larger the value of ES, the better the image fusion performance.
2. Spatial frequency (SF) measures the number of details presented in a stimulus per degree of visual angle, and can be given as follows:
  (14) $$SF=\sqrt{RF^{2}+CF^{2}},\;$$
  (15) $$RF=\sqrt{\frac{1}{MN}\sum_{i=0}^{M-1}\sum_{j=1}^{N-1}{\left[Z\left(i,j\right)-Z\left(i,\left(j-1\right)\right)\right]}^{2}},$$
  (16) $$CF=\sqrt{\frac{1}{MN}\sum_{i=1}^{M-1}\sum_{j=0}^{N-1}{\left[Z\left(i,j\right)-Z\left(\left(i-1\right),j\right)\right]}^{2}},$$
  where RF and CF represent the row frequency and column frequency, respectively, and $Z\left(i,j\right)$ denotes the gray-value intensity of the pixel located at $\left(i,j\right)$ in the image. A higher SF value of an image meant that it contained more details—and hence, led to a better fusion result.
3. Standard deviation (SD) is the square root of the variance, which refers to the image contrast. The higher the contrast, the greater the value of SD. SD was calculated as follows:
  (17) $$SD=\sqrt{\frac{1}{MN}\sum_{i=1}^{M}\sum_{j=1}^{N}{\left(Z\left(i,j\right)-\dot{\mu }\right)}^{2}},\;$$
  where $\dot{\mu }$ stands for the mean intensity of the image.
4. Entropy (H) [37] measures how much information is contained in an image, calculated as follows:
  (18) $$H=-\sum_{l=0}^{L-1}{\bar{p}}_{l}{log}_{2}\left(p_{l}\right),$$
  where L represents the gray level of an image and $\bar{p_{l}}$ stands for the probability of the lth gray level in the image. A larger H value signified a better image fusion performance.
5. Feature mutual information (FMI) [38,39] refers to how much feature information is successfully transferred from the original images ($I_{AC}$, $I_{DPC}$, and $I_{DFC}$) to the fused image $I_{F}$, mathematically defined as follows:
  (19) $$FMI=FI\left(I_{AC},I_{F}\right)+FI\left(I_{DPC},I_{F}\right)+FI\left(I_{DFC},I_{F}\right),\;$$
  where $FI\;\left(I_{A},I_{B}\right)$ stands for the amount of feature information transferred from image $I_{A}$ to image $I_{B}$; FI, in Formula (19), can be calculated as follows:
  (20) $$FI\left(I_{AC},I_{F}\right)=\sum_{I_{AC},I_{F}}\left[\;p_{I_{AC},I_{F}}\left(i,j,k,l\right){log}_{2}\frac{p_{I_{AC},I_{F}}\left(i,j,k,l\right)}{p_{I_{AC}}\left(i,j\right)p_{I_{F}}\left(k,l\right)}\right],\;$$
  (21) $$FI\left(I_{DPC},I_{F}\right)=\sum_{I_{DPC},I_{F}}\left[\;p_{I_{DPC},I_{F}}\left(i,j,k,l\right){log}_{2}\frac{p_{I_{DPC},I_{F}}\left(i,j,k,l\right)}{p_{I_{DPC}}\left(i,j\right)p_{I_{F}}\left(k,l\right)}\right],$$
  (22) $$FI\left(I_{DFC},I_{F}\right)=\sum_{I_{DFC},I_{F}}\left[\;p_{I_{DFC},I_{F}}\left(i,j,k,l\right){log}_{2}\frac{p_{I_{DFC},I_{F}}\left(i,j,k,l\right)}{p_{I_{DFC}}\left(i,j\right)p_{I_{F}}\left(k,l\right)}\right],$$
  where $p_{A,B}$ is the joint distribution function between image A and image B, and $\left(i,j\right)$ and $\left(k,l\right)$ denote the pixel coordinates in image A and image B, respectively. Should the value of FMI be more significant, the fusion scheme fused three images successfully, preserving more feature information from each image.
6. The feature similarity index measure (FSIM) [40,41] related to the similarity between two images based on the low-level features—specifically, the phase congruency (PC) and the image gradient magnitude (GM). The FSIM of two images, $I_{A}\left(i,j\right)$ and $I_{B}\left(i,j\right)$, were calculated by:
  (23) $$FSIM\left(A,B\right)=\frac{\sum_{i=1}^{M}\sum_{j=1}^{N}S_{AB}\left(i,j\right)max\left[PC_{A}\left(i,j\right),PC_{B}\left(i,j\right)\right]}{\sum_{i=1}^{M}\sum_{j=1}^{N}max\left[PC_{A}\left(i,j\right),PC_{B}\left(i,j\right)\right]},\;$$
  where $PC_{A}$ and $PC_{B}$ are the PC values of $I_{A}$ and $I_{B}$, respectively, and $S_{AB}\left(i,j\right)$ refers to the local similarity, denoted as follows:
  (24) $$S_{AB}\left(i,j\right)={\left[S_{PC;AB}\left(i,j\right)\right]}^{\alpha }{\left[S_{GM;AB}\left(i,j\right)\right]}^{\beta },\;$$
  (25) $$S_{PC;AB}\left(i,j\right)=\frac{2PC_{A}\left(i,j\right)PC_{B}\left(i,j\right)+T_{1}}{2PC_{A}{}^{2}\left(i,j\right)PC_{B}{}^{2}\left(i,j\right)+T_{1}},$$
  (26) $$S_{GM;AB}\left(i,j\right)=\frac{2GM_{A}\left(i,j\right)GM_{B}\left(i,j\right)+T_{2}}{2GM_{A}{}^{2}\left(i,j\right)GM_{B}{}^{2}\left(i,j\right)+T_{2}},$$
  where $S_{PC;AB}\left(i,j\right)$ and $S_{GM;AB}\left(i,j\right)$ are similarity measurements for $I_{A}\left(i,j\right)$ and $I_{B}\left(i,j\right),$ based on PC and GM respectively; α and β are two parameters; and $T_{1}$ and $T_{2}$ are two constants, all of which were defined in [36]. To measure the performance of the XGI fusion, the overall FSIM was calculated by averaging $FSIM\left(I_{AC},I_{F}\right)$, $FSIM\left(I_{DPC},I_{F}\right)$, and $FSIM\left(I_{DFC},I_{F}\right)$, where $I_{F}$ denoted the fusion result. The higher the FSIM value, the better the fusion performance.
7. The fusion factor (FF) is based on mutual information (MI), which originally measures the statistical dependence between two random variables as a concept in information theory. It is capable of measuring how much information was transferred from the input image to the fused image, and was defined as follows:
  (27) $$FF=MI\left(I_{AC},I_{F}\right)+MI\left(I_{DPC},I_{F}\right)+MI\left(I_{DFC},I_{F}\right),$$
  where
  (28) $$MI\left(I_{AC},I_{F}\right)=\sum_{I_{AC},I_{F}}\left[\overset{=}{P}\left(I_{AC},I_{F}\right)log\frac{\overset{=}{P}\left(I_{AC},I_{F}\right)}{\overset{=}{P}\left(I_{AC}\right)\overset{=}{P}\left(I_{F}\right)}\right],$$
  (29) $$MI\left(I_{DPC},I_{F}\right)=\sum_{I_{DPC},I_{F}}\left[\overset{=}{P}\left(I_{DPC},I_{F}\right)log\frac{\overset{=}{P}\left(I_{DPC},I_{F}\right)}{\overset{=}{P}\left(I_{DPC}\right)\overset{=}{P}\left(I_{F}\right)}\right],$$
  (30) $$MI\left(I_{DFC},I_{F}\right)=\sum_{I_{DFC},I_{F}}\left[\overset{=}{P}\left(I_{DFC},I_{F}\right)log\frac{\overset{\cdot }{P}\left(I_{DFC},I_{F}\right)}{\overset{\cdot }{P}\left(I_{DFC}\right)\overset{\cdot }{P}\left(I_{F}\right)}\right],$$
  where $MI\left(I_{AC},I_{F}\right)$, $MI\left(I_{DPC},I_{F}\right)$, and $MI\left(I_{DFC},I_{F}\right)$ refer to the mutual information between images $I_{AC}$ and $I_{F}$, $I_{DPC}$ and $I_{F}$, and $I_{DFC}$ and $I_{F}$, respectively; $\overset{=}{P}\left(I_{A},I_{B}\right)$ is the joint probability density function of two images; and $\overset{=}{P}\left(I_{A}\right)$ is the probability density function of an image. A larger FF value means a better image fusion performance.
8. The structural similarity index measure (SSIM) [42] measures how much structural information was transferred from one image into another based on the human eye’s sensitivity to the structural information, given as follows:
  (31) $$SSIM\left(I_{A},I_{B}\right)=\frac{\sum_{j=1}^{W}SSIM\left(I_{A}{}_{j},I_{B}{}_{j}\right)}{W},$$
  where $SSIM\left(I_{A},I_{B}\right)$ represents the SSIM value of images $I_{A}$ and $I_{B}$; W is the number of windows that come from the division of an image; and $SSIM\left(I_{A}{}_{j},I_{B}{}_{j}\right)$ denotes the structural similarity between images $I_{A}$ and $I_{B}$ in the jth window. This was calculated by:
  (32) $$SSIM\left(I_{A}{}_{j},I_{B}{}_{j}\right)=\frac{\left(2\mu_{I_{A}{}_{j}}\mu_{I_{B}{}_{j}}+k_{1}{}^{2}L^{2}\right)\left(2\sigma_{I_{A}{}_{j}I_{B}{}_{j}}+k_{2}{}^{2}L^{2}\right)}{\left(\mu_{I_{A}{}_{j}}{}^{2}+\mu_{I_{B}{}_{j}}{}^{2}+k_{1}{}^{2}L^{2}\right)\left(\sigma_{I_{A}{}_{j}}{}^{2}+\sigma_{I_{B}{}_{j}}{}^{2}+k_{2}{}^{2}L^{2}\right)},$$
  where $\mu_{I_{A}{}_{j}}$, $\mu_{I_{B}{}_{j}}$, $\sigma_{I_{A}{}_{j}}{}^{2}$, and $\sigma_{I_{B}{}_{j}}{}^{2}$ are the local means and the local variances of the jth windows in images $I_{A}$ and $I_{B}$, respectively; $\sigma_{I_{A}{}_{j}I_{B}{}_{j}}{}^{2}$ is the cross-covariance for the jth windows between $I_{A}$ and $I_{B}$. An overall SSIM value for the XGI fusion was defined as follows:
  (33) $$SSIM=\frac{SSIM\left(I_{AC},I_{F}\right)+SSIM\left(I_{DPC},I_{F}\right)+SSIM\left(I_{DFC},I_{F}\right)}{3},$$
  where $I_{AC}$, $I_{DPC}$, $I_{DFC}$, and $I_{F}$ denote the three input images and the fused image, respectively. Note that larger SSIM values corresponded to better fusion performance.
9. Power spectral density (PSD) [43,44] measures the power at each signal frequency. The estimate of the PSD $P_{j}$ at frequency j was denoted as follows:
  (34) $$P_{j}={\left(\frac{\left|C_{j}\right|}{n}\right)}^{2},\;$$
  where $C_{j}$ are the Fourier terms and n is the number of samples. The total area enclosed by the PSD curve and the coordinate axis denoted the information contained in an image. The PSD curve of one image within one frequency band was higher than that of the other image, which meant that the former image had more information in this frequency band. A generally higher PSD curve indicated a better image fusion performance [42].

## 5. Experiment

### 5.1. Image Fusion Parameters and Results

The fusion parameters used in this work were given by the order of the fusion steps. For step 1, the sizes of the neighborhood samples for adaptive Wiener filtering were set at $\left[5,\;5\right]$. For step 2, the decomposition levels of NSCT were $\left[4,\;4,\;4,\;4\right]$. With regard to the parameters of the SCM, defined in Equations (4)–(6), we empirically set f=0.8, g=0.7, h=20, $W=\left[0.1091,\;0.1409,\;0.1091;\;0.1409,\;0,\;0.1409;\;0.1091,\;0.1409,\;0.1091\right]$, and the total iterative counts k=200. Weight factor for low-frequency band: a=0.55. Weight factors for high-frequency bands: b=0.41, c=0.29, d=0.30, and Tth=1. For step 3, the number of tiles, by row and column, used for CLAHE was $\left[5,\;5\right]$, the contrast enhancement limit parameters for CLAHE were $\left[0,\;1\right]$ and 0.00125, and the CLAHE histogram’s number of bins was 500. Finally, λ1 and λ2 for contrast optimization were 4.8 and 0.49, respectively.

**Figure 4:**
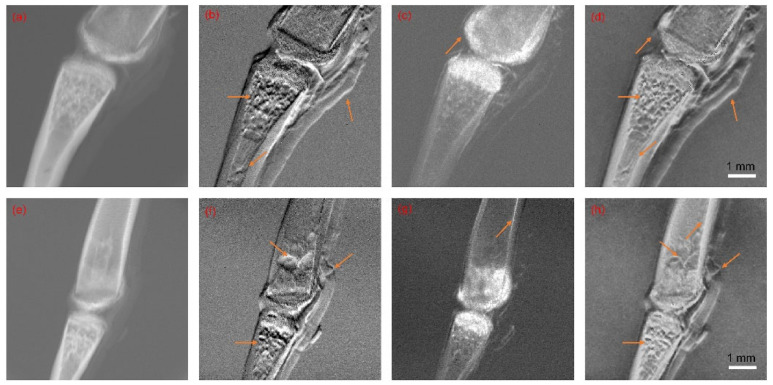
Source images and fusion results. (**a**,**e**) Source images from the AC channel; (**b**,**f**) source images from the DPC channel; (**c**,**g**) source images from the DFC channel; (**d**,**h**) fusion results by NSCT-SCM. The orange arrows point out distinct differences between tri-contrast modalities.

The data used for the fusion process came from the grating-based X-ray phase contrast imaging of frog toes [45]. These images (a total of four sets of images) were fused by our algorithm using the parameters above. These experiments were carried out on MATLAB and half the results (of two sets of images) are shown in Figure 4. The remaining results of the other two sets of images are given in the Supplemental Information Section S2.

As shown in Figure 4, many features that only appeared in the DPC or DFC channels were successfully transported to the final fusion results. The soft tissue around the bone and meshwork structure of the bone trabecula (which can only be observed in the DPC channel), as well as the high signal of the bone cortex (which is only visible in the DFC channel), were successfully transferred into the fusion results. These well-preserved features demonstrated the efficiency of the proposed fusion scheme.

### 5.2. Objective Evaluation and Discussion

**Figure 5:**
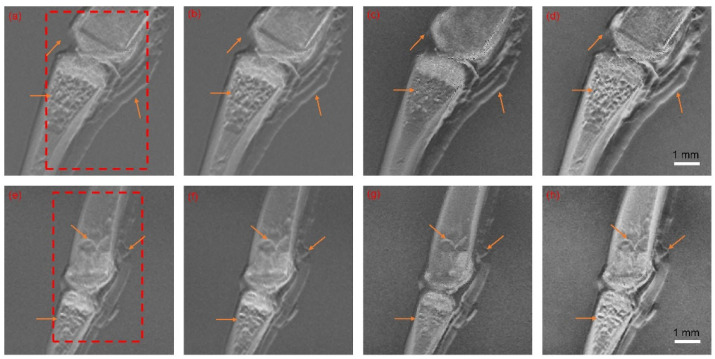
Fusion results of (**a**,**e**) NSCT, (**b**,**f**) NSCT-PCNN, (**c**,**g**) SIDWT, and (**d**,**h**) the proposed method (NSCT-SCM). The red boxes denote the region of interest used to calculated objective evaluation criteria. The orange arrows point out distinct differences between results of image fusion methods.

In this section, we implemented the other three image fusion schemes on the same datasets as those in Section 5.1. These methods included the algorithm based on the shift-invariance discrete wavelet transform (SIDWT) [17], the traditional NSCT image fusion algorithm, and the conventional NSCT-PCNN image fusion algorithm [46]. Then, the performance results of all four methods were evaluated by the measures mentioned in Section 4. Half of the results (of two sets of images) are displayed in Figure 5 and Table 1 and Table 2, while the remaining results of the other 2 datasets are given in the Supplemental Information, Section S2.

With regard to the parameter settings of SIDWT, the size of the neighborhood samples used for adaptive Wiener filtering was $\left[5,\;5\right]$; the decomposition levels of the first and second fusion steps were 4 and 5, respectively; the numbers of tiles by row and column used for CLAHE were $\left[5,\;5\right]$; the limit of CLAHE contrast enhancement was $\left[0,1\right]:\;0.0017$; the CLAHE histogram’ number of bins was 500; and $\lambda_{1}$ and $\lambda_{2},$ for he contrast optimization, were 3.9 and 0.59, respectively. The parameter settings of the NSCT used in the NSCT-PCNN method and NSCT method were the same as those we mentioned in Section 5.1. In addition, the parameters of the PCNN were empirically set as follows: $\alpha_{L}=0.06931$, $\alpha_{\theta }=0.2$, $V_{L}=1$, $V_{\theta }=20$, θ=0.2, N=200, and linking weight $W=\left[0.707,1,0.707;1,0,1;0.707,1,0.707\right]$ [46].

As shown in Figure 5, we marked areas with red squares, called the regions of interest (ROI), to reduce the impact of noise on evaluation and focus on the part of the image in which we were most interested. We observed that the soft tissue around the bone was better presented by the NSCT-SCM methods than others. Our proposed method also preserved the texture inside the bones and the details at the bone joint junctions. In contrast, the details and texture of the other methods were not satisfactory, with images that were blurrier and less sharp in comparison, indicating those methods’ tendency to compromise on information preservation. The objective evaluation criteria were further carried out on these fusion results, and the evaluation results of the ROI are given in Table 1 and Table 2. The best results for each measure are marked in bold.

As shown in Table 1 and Table 2, the results of FMI, FF, SSIM, and FSIM of all methods were at the same level, with some slight fluctuations. This indicated that all methods demonstrated the ability to output fusion results that were similar enough to the source images. However, regarding the outcomes of ES, H, SD, and SF, the proposed method generally outperformed the others, showing that NSCT-SCM was able to transfer more information and details from the source images to the fusion result than other methods. Specifically, NSCT-SCM had higher values regarding H, SD, and SF in Table 1 and H and SD in Table 2. The NSCT method also led to the best ES results, as shown in in Table 1. The NSCT-PCNN method outperformed others, with regard to ES, and the SIDWT showed the best SF value.

**Figure 6:**
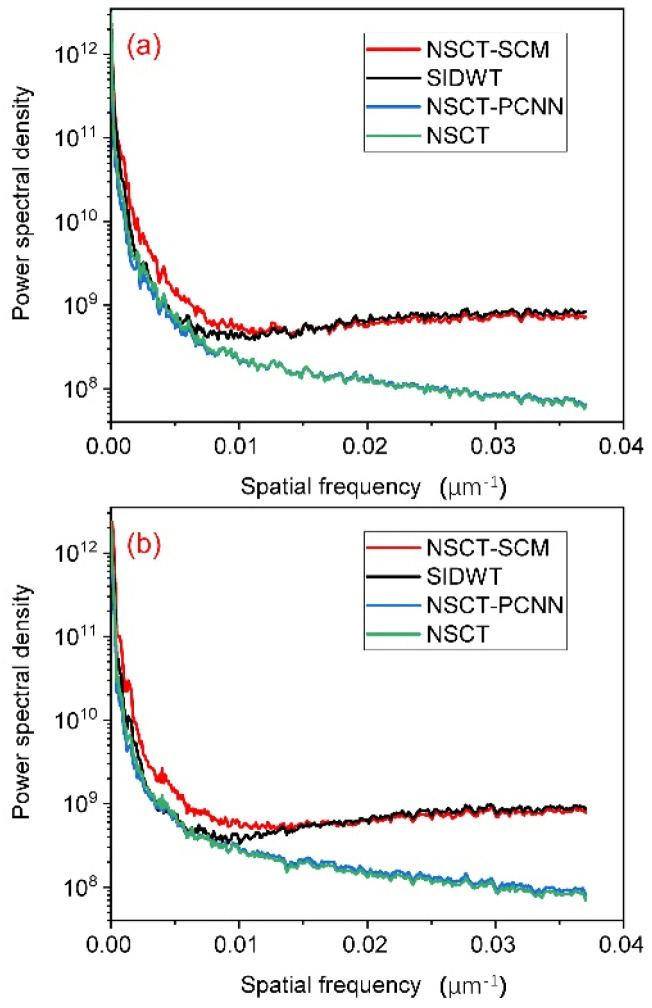
PSD curves of (**a**) Figure 5a–d and (**b**) Figure 5e–h.

In addition, we calculated the PSD of each fusion result and drew the PSD curves of the fusion images, given in Figure 6.

As shown in Figure 6, the PSD curve of our proposed scheme was generally higher than the others, meaning that the fusion results of NSCT-SCM contained more information and were of better quality. In addition, although the power spectral density of the SIDWT remained at the same level as that of the proposed method in high spatial frequencies, it was significantly outperformed by the NSCT-SCM in low spatial frequencies. This result was consistent with the evaluation results of the above eight measures and the subjective evaluation results, i.e., that the fusion image of NSCT-SCM had higher contrast and finer details.

## 6. Conclusions

In the present work, an NSCT-SCM-based image fusion scheme was proposed for X-ray grating interferometry. It incorporated three major steps: denoising, the NSCT-SCM fusion algorithm, and enhancement. A new coefficient selection strategy was proposed for the fusion algorithm step, which selected coefficients in different ways concerning high-frequency and low-frequency coefficients. This strategy met a unique requirement of XGI: that the low-frequency coefficient should derive primarily from the AC channel in order to achieve final fusion results similar to traditional CT, and that the high-frequency coefficient should be selected in a way preserves the details and features in the DPC and DFC channels.

Furthermore, the proposed method and three other image fusion methods were implemented on X-ray grating interferometry data of frog toes to demonstrate the feasibility and robustness of the NSCT-SCM image fusion scheme. The fusion results were evaluated using both subjective and objective measures. As observed and demonstrated, the proposed method was competitive with the other image fusion methods, both visually and quantitatively. The proposed image fusion scheme output images with high contrast and explicit details, and demonstrated the potential for real-time application. In our future research, a feature-based fusion scheme will be studied to process images more similarly to human eyes and achieve better computational efficiency.

## Figures and Tables

**Table 1:** The evaluation results of the ROI in Figure 5a–d.

Measures	NSCT	NSCT-PCNN	SIDWT	Proposed Method (NSCT-SCM)
ES	**2.6297**	2.2885	0.6527	1.8847
H	5.8758	5.6990	6.5755	**7.0350**
SD	0.0962	0.0830	0.1229	**0.1615**
SF	12.1136	14.0702	40.3987	**40.6443**
FMI	**0.9524**	**0.9524**	0.9181	0.9321
FF	13.1018	13.0406	12.9649	**13.4200**
SSIM	0.9973	0.9970	**0.9974**	0.9961
FSIM	**0.9390**	0.9381	0.9304	0.9234

**Table 2:** The evaluation results of the ROI in Figure 5e–h.

Measures	NSCT	NSCT-PCNN	SIDWT	Proposed Method (NSCT-SCM)
ES	1.2587	**1.1371**	0.3937	1.1191
H	6.0928	6.2928	6.9253	**7.2230**
SD	0.1077	0.1077	0.1471	**0.1821**
SF	8.3268	8.3268	**30.0311**	24.2106
FMI	0.9336	**0.9936**	0.8545	0.8943
FF	13.7133	13.7133	13.5084	14.2617
SSIM	**0.9974**	**0.9974**	0.9964	0.9968
FSIM	**0.9368**	**0.9368**	0.9214	0.9318

## Data Availability

The datasets generated during and/or analyzed during the current study are available from the corresponding author on reasonable request.

## Supplementary Materials

The following supporting information can be downloaded at: https://www.mdpi.com/article/10.3390/s23063115/s1.
