# Supplementary material for: Multimodal Image Fusion for X-ray Grating Interferometry
Source: Sensors (Basel). 2023 Mar 14;23(6):3115. doi: 10.3390/s23063115 (PMC10053574; doi:10.3390/s23063115)
Supplement: Supplementary file 1 [file sensors-23-03115-s001.zip › sensors-2205766-supplementary.pdf]

Multimodal Image Fusion for X-ray Grating Interferometry

Haoran Liu [1,2], Mingzhe Liu [1,2,*], Xin Jiang [1], Jinglei Luo [3], Yuming Song [3], Xingyue Chu [3], Guibin Zan [4,*]

[1] School of Data Science and Artificial Intelligence, Wenzhou University of Technology, Wenzhou 325000, China
[2] State Key Laboratory of Geohazard Prevention and Geoenvironment Protection, Chengdu University of Technology, Chengdu 610059, China
[3] The Engineering & Technical College of Chengdu University of Technology, Leshan 614000, China
[4] Sigray, Inc., 5750 Imhoff Drive, Concord, CA 94520, United States of America
* Correspondence: authors, liumz@cdut.edu.cn (M.L.), gbzan@sigray.com (G.Z.)

Section S1 Programming scheme of the high-frequency fusion rule

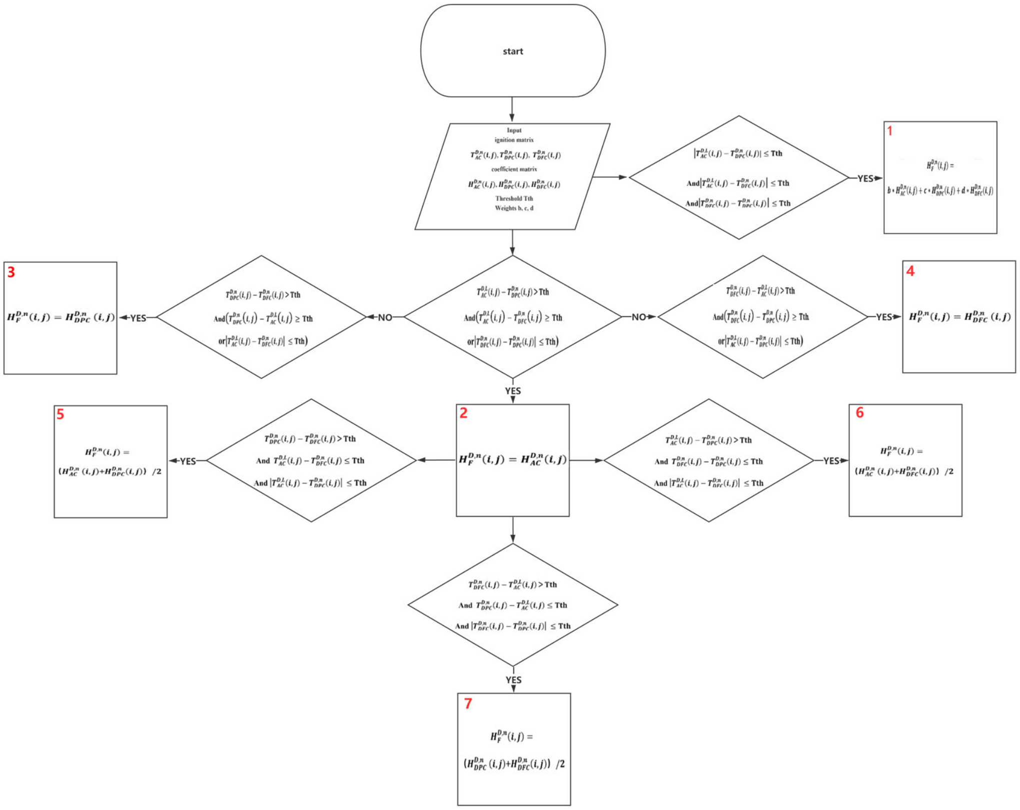

Figure S1. The flow chat of the high-frequency fusion rule.

As shown in Fig.S1, there are totally 7 possible values for the $H_F^{D,n}(i,j)$, which are 1. $H_F^{D,n}(i,j) = b \cdot H_{AC}^{D,n}(i,j) + c \cdot H_{DPC}^{D,n}(i,j) + d \cdot H_{DFC}^{D,n}(i,j)$, 2. $H_F^{D,n}(i,j) = H_{AC}^{D,n}(i,j)$, 3. $H_F^{D,n}(i,j) = H_{DPC}^{D,n}(i,j)$, 4. $H_F^{D,n}(i,j) = H_{DFC}^{D,n}(i,j)$, 5. $H_F^{D,n}(i,j) = (H_{AC}^{D,n}(i,j) + H_{DPC}^{D,n}(i,j))/2$, 6. $H_F^{D,n}(i,j) = (H_{AC}^{D,n}(i,j) + H_{DFC}^{D,n}(i,j))/2$, and 7. $H_F^{D,n}(i,j) = (H_{DPC}^{D,n}(i,j) + H_{DFC}^{D,n}(i,j))/2$ respectively. The programming idea of this flow chart is that we set a threshold Tth for the comparison of ignition results $T_{AC}^{D,L}$, $T_{DPC}^{D,L}$ and $T_{DFC}^{D,L}$, which is used to measure whether the information of a pixel coming from a single channel is significant enough to replace the others or one needs to weighted average the information of two or three channels. To be specific, when one among them is significantly bigger than others, we chose the coefficient from this channel as the value of the $H_F^{D,n}(i,j)$ directly; when two among them are significantly bigger than the rest one, we take the average of them as the value of the $H_F^{D,n}(i,j)$; and when none of them is significantly bigger than others, we weighted average

the value of all three channels as the value of the $H_F^{D,n}(i,j)$ by the weight factor b, c and d.

Section S2 Other 2 sets of experimental results

To conform with the total words and images limitation, we just showed part of our experimental results in the text of our work. The rest of them are shown in this section.

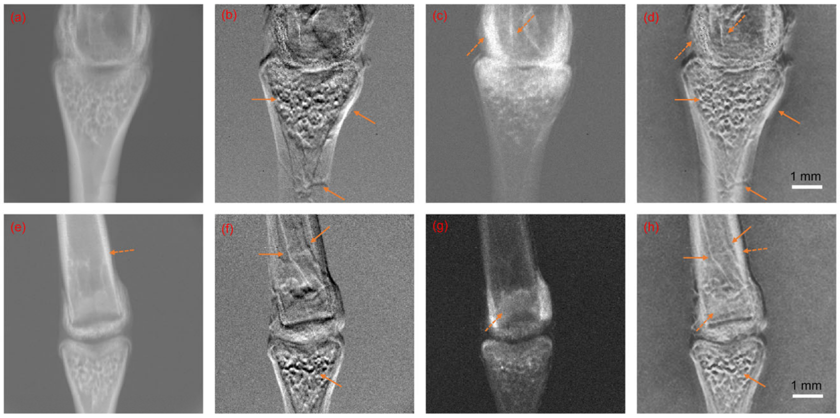

Figure S2. The source images and fusion results, (**a**,**e**)the source images from the AC channel, (**b**,**f**)the source images from the DPC channel, (**c**,**g**) the source images from the DFC channel, (**d**,**h**) the fusion results by NSCT-SCM.

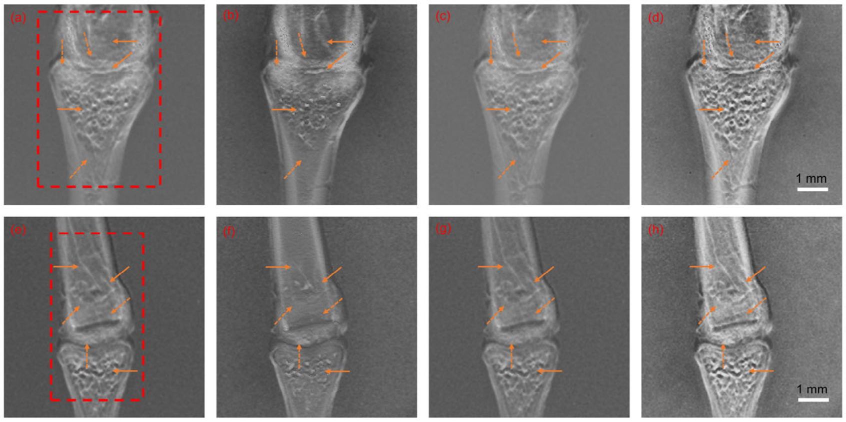

Figure S3. The fusion results of four different methods. (**a**,**e**) the fusion results of the NSCT, (**b**,**f**) the fusion results of the NSCT-PCNN, (**c**,**g**) the fusion results of the SIDWT, (**d**,**h**) the fusion results of the proposed method (NSCT-SCM).

Table S1. The evaluation results of four methods for the ROI of Fig. 8 (a)-(d).

Measures	NSCT	NSCT-PCNN	SIDWT	Proposed method (NSCT-SCM)
$\boldsymbol{ES}$	1.4638	**1.6501**	0.3920	1.2237
$\boldsymbol{H}$	6.0681	6.2201	6.7266	**7.0965**
$\boldsymbol{SD}$	0.0871	0.0981	0.1110	**0.1427**
$\boldsymbol{SF}$	7.4900	6.4478	24.3723	**25.2588**
$\boldsymbol{FMI}$	0.9369	**0.9373**	0.8617	0.8936
$\boldsymbol{FF}$	13.4804	**13.5711**	**13.5711**	13.4804
$\boldsymbol{SSIM}$	0.9976	**0.9979**	0.9977	0.9976

FSIM	0.9280	**0.9393**	0.9280	0.9280

Table S2. The evaluation results of four methods for the ROI of Fig. 5 (e)-(h).

Measures	NSCT	NSCT-PCNN	SIDWT	Proposed method (NSCT-SCM)
ES	**0.7692**	0.7193	0.2234	0.6109
H	6.4868	6.2727	6.7961	**7.2363**
SD	0.1180	0.0969	0.1206	**0.1735**
SF	7.0437	8.3599	**27.1564**	24.2602
FMI	0.9422	**0.9464**	0.8624	0.8942
FF	13.500	13.5344	13.3926	**14.1994**
SSIM	0.9957	0.9957	**0.9960**	0.9957
FSIM	**0.9432**	0.9416	0.9229	0.9312

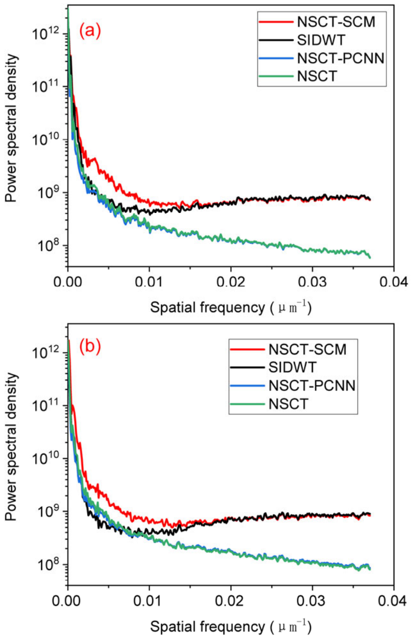

Figure S4. The PSD curves of fusion images, (**a**) the PSD curves of Figure S3 (a–d), (**b**) the PSD curves of Figure S3 (e–h).
